# Breath Gas Markers in Depression and Their Relationship with Brain Metabolism

**DOI:** 10.1192/j.eurpsy.2024.1115

**Published:** 2024-08-27

**Authors:** D. Keskin Gokcelli, J. Itzhacki Sitton, J. Kesik, D. H. Henning, E. Farrher, N. J. Shah, T. Frodl

**Affiliations:** ^1^Department of Psychiatry, RWTH Aachen University, Aachen; ^2^Institute of Neuroscience and Medicine 4, Forschungszentrum Jülich, INM-4, Jülich; ^3^Department of Neurology; ^4^JARA – BRAIN – Translational Medicine, RWTH Aachen University, Aachen, Germany

## Abstract

**Introduction:**

Dysfunctional changes in the glutamatergic system play an important role in the pathophysiology of depression. Glutamate regulates various neuronal function, such as nerve migration, excitability, plasticity, as well as long-term potentiation and long-term synaptic depression. Failures in this process might cause emotional/cognitive changes associated with stress-induced depressive symptoms, a part of our current understanding of the pathophysiology of depression. These changes might be related to deviations in biochemical blood parameters, but also to volatile organic compounds (VOCs) measured in breath.

**Objectives:**

1) To replicate our previous finding that concentration of volatile organic compounds in expiratory breath gas and metabolites derived from MR spectroscopy distinguish unmedicated depressed patients from healthy participants, (2) to determine whether the amount of these VOCs is associated with severity of depression and anxiety, and (3) to correlate breath-VOC-content with glutamatergic neurotransmission and energy metabolism derived from MR spectroscopy.

**Methods:**

25 antidepressant-free patients with major depression according to DSM V (18-65 years of age) are recruited from our out- and inpatient clinics. The controls will consist of 25 healthy age-and-sex-matched participants. Breath gas analyses will be carried out at awakening, and 30 and 60 minutes thereafter, and at 5pm using PTR-TOF-MS with direct on time measurement through a special sampler. A 7 Tesla Siemens Terra MRI scanner will be used to undertake spectroscopic measurements. Concentrations of glutamate and β-hydroxybutyrate levels in the pregenual and dorsal anterior cingulate gyrus will subsequently be assessed.

**Results:**

Statistical analysis for differences between groups corrected for multiple measurements will be carried out. Concentration of VOCs will be correlated with brain metabolism and severity of symptoms.

**Conclusions:**

VOCs in breath are proposed to be an efficient and non-invasive marker for depression-related biochemical changes related to disease severity, and eventually useful for personalized treatment planning.

**Disclosure of Interest:**

None Declared

